# MicroRNA as a novel player in atrial fibrillation

**DOI:** 10.3389/fgene.2014.00097

**Published:** 2014-04-30

**Authors:** Siyi Fu, Leqi Huang, Yalong Wang, Xing Li, Jie Li, Junjie Xiao

**Affiliations:** ^1^Regeneration Lab and Experimental Center of Life Sciences, School of Life Science, Shanghai UniversityShanghai, China; ^2^Shanghai Key Laboratory of Bio-Energy Crops, School of Life Sciences, Shanghai UniversityShanghai, China; ^3^Innovative Drug Research Center of Shanghai UniversityShanghai, China

**Keywords:** microRNA, atrial fibrillation, electrial remodeling, structural remodeling, therapy

Atrial fibrillation (AF), with an extremely highly age-dependent prevalence, is a most frequent type of sustained arrhythmia in the clinic both in developing and developed countries (Dobrev and Nattel, 2011). AF patients have a lot of symptoms including palpitations, dizziness, breathlessness, and chest pain. AF will lead to an increased risk of stroke and aggravate congestive heart failure (Shi et al., 2013). Compared with familial AF, the non-familial AF occupies the majority of AF. In China, at least 10 million people are affected by it. Therefore, AF is associated with a remarkable morbidity and mortality, leading to a large socio-economic burden. Unfortunately, the underling mechanisms of AF are still exclusive (Wang et al., 2011; Liu et al., 2012a,b).

Several hypotheses have been proposed for AF. Focal activity, single-circuit reentry, and multiple-circuit reentry have been generally considered as three classic models for AF. In addition, different substrates which facilitate the formation of AF have also been revealed including electrical remodeling, structure remodeling, and intracellular Ca^2+^ handling remodeling. Moreover, various genetic mutations such as chromosomal loci (10q22-q24 and 6q14-16) mutations, ion channel (Ka^+^ and Na^+^) mutations, connexin (GJA5 and GJA1) mutations in the familial AF and more recently several common variants on 4q25, 16q22, and 1q21 in the non-familial AF have been identified (Xiao et al., 2011a,b; Liu et al., 2012a,b). Despite this, the definitive elucidation of AF still remains relatively limited and unclear (Zhang et al., 2011).

MicroRNAs (miRNAs, miRs) are a novel class of endogenous non-coding RNAs of around 22 nucleotides in length, which are widely accepted to possess a key role in the gene expression regulatory network at the post-transcriptional level (Wang et al., 2011; Fu et al., 2013). So far, at least one thousand miRNAs have been identified and they have been reported to participate in many fundamental biological processes including cell proliferation, growth, differentiation, apoptosis, and tissue remodeling. Dysregulated miRNAs have been shown to be related to the genesis of many cardiovascular diseases, including arrhythmia, hypertrophy, and heart failure. Moreover, miRNAs have also been identified to be necessary for the differentiation of human-derived cardiomyocyte progenitor cells (Xiao et al., 2012). Furthermore, circulating miRNAs can also be served as promising biomarkers for acute myocardial infarction and heart failure etc. (Dimmeler and Zeiher, 2010; Li et al., 2013). Thus, it is not surprising that miRNAs gain a critical position in the physiological and pathological processes of the cardiovascular system (Wang et al., 2011; Xiao et al., 2011a,b; Liu et al., 2012a,b).

Several studies have presented interesting connections between miRNAs and AF. Of note, a group of miRNAs has been identified to regulate target genes encoding cardiac ion channels/transporters/Ca^2+^-handling proteins, which may participate in the genesis of AF (Wang et al., 2011; Dobrev, 2012). Interestingly, many available data have demonstratedthatmiR-1, miR-21, miR-26, miR-29, miR-30, miR-133, miR-208, miR-328, miR-499, and miR-590 might take part in the genesis of AF (Dawson et al., 2013; Shi et al., 2013). Most of these miRNAs promote the electrical or structural remodeling in the atrium. MiR-26 family contributes to AF via repressing the expression of KCNJ2/Kir2.1/I_K1_ while miRNA-1 via regulating I_K1_ expression and Ca^2+^ handling proteins. In addition, miR-499 regulates KCNN3/SK3 while miR-328 targets CACNA1C and CACNB, contributing to adverse electrical remodeling (Lu et al., 2010). Moreover, miR-133 and miR-590 have been found to target transforming growth factor-β 1 (TGF-β 1) and TGF-β receptor type II (TGF-β RII), leading to structure remodeling in AF (Shan et al., 2009). Interestingly, circulating levels of miR-328 and miR-150 have been reported to be down-regulated in AF patients though the functional role is still unclear (Liu et al., 2012a,b; McManus et al., 2014).

With more systematic and insightful studies exploring the miRNAs basis for AF, novel therapeutics will be developed and ultimately lead to advanced treatment (Zhao et al., 2013).

## Conflict of interest statement

The authors declare that the research was conducted in the absence of any commercial or financial relationships that could be construed as a potential conflict of interest.
